# Complete radiologic and clinical reversal of lumbar spinal epidural lipomatosis via GLP-1 agonist

**DOI:** 10.1093/jscr/rjag052

**Published:** 2026-02-12

**Authors:** Lennard M Wurm, Peter Koch, Wolfgang Ertel, Dominik Laue

**Affiliations:** Department of Traumatology and Reconstructive Surgery, Charité, Universitätsmedizin Berlin, corporate member of Freie Universität Berlin, Humboldt-Universität zu Berlin and Berlin Institute of Health, Berlin, Germany; Medical Faculty Heinrich-Heine University and University Hospital Düsseldorf, Düsseldorf, Germany; Department of Neurology, Private Practice, Berlin, Germany; Department of Traumatology and Reconstructive Surgery, Charité, Universitätsmedizin Berlin, corporate member of Freie Universität Berlin, Humboldt-Universität zu Berlin and Berlin Institute of Health, Berlin, Germany; Department of Traumatology and Reconstructive Surgery, Charité, Universitätsmedizin Berlin, corporate member of Freie Universität Berlin, Humboldt-Universität zu Berlin and Berlin Institute of Health, Berlin, Germany

**Keywords:** spinal epidural lipomatosis, GLP-1 receptor agonist, Semaglutide, lumbar spinal stenosis, medical weight loss, medical cannabis

## Abstract

Spinal epidural lipomatosis (SEL) is a rare cause of lumbar canal stenosis, typically linked to obesity or corticosteroid use. Severe cases often require decompression. We report a 48-year-old man with chronic low back pain and morbid obesity (153 kg) who presented with extensive SEL and critical stenosis. Previous conservative therapies failed. A non-surgical strategy using GLP-1–induced weight loss (semaglutide) and medical cannabis for pain control was initiated. Over a follow-up of one year, his weight dropped to 93.8 kg, with major improvement in pain and mobility. MRI demonstrated near-complete regression of epidural fat and resolution of stenosis, avoiding surgery. The patient remained neurologically intact. This appears to be the first documented case of SEL reversal achieved through medical weight loss alone. The findings highlight GLP-1 agonists as a potential therapeutic option in obesity-related spinal disorders and support cannabis as part of non-opioid pain management.

## Introduction

Lumbar spinal epidural lipomatosis (SEL) is an uncommon cause of lumbar spinal canal stenosis, characterized by pathological overgrowth of unencapsulated epidural fat. SEL is most frequently associated with chronic corticosteroid exposure or morbid obesity, though idiopathic cases occur [1, 2]. Patients typically present with low back pain, neurogenic claudication, or radiculopathy; in advanced cases, cauda equina syndrome may develop. Management ranges from weight reduction and steroid withdrawal to surgical decompression. In practice, many patients ultimately undergo laminectomy or epidural fat debulking, but perioperative risks are considerable [3, 4].

Weight loss is the cornerstone of conservative therapy in obesity-related SEL. Case reports suggest that both bariatric surgery and nonsurgical weight loss can lead to radiographic and clinical improvement, indicating that epidural fat is metabolically responsive and that reducing systemic adiposity can decompress the spinal canal [5].

Glucagon-like peptide-1 (GLP-1) receptor agonists have recently emerged as effective pharmacologic therapy for obesity, inducing relevant weight loss with mainly transient gastrointestinal side effects. This makes GLP-1 agonists attractive for high-risk obese patients in whom surgery carries substantial risk [6]. These agents mimic the endogenous incretin hormone GLP-1, promoting glucose-dependent insulin secretion and suppressing glucagon. Crucially for weight loss, they delay gastric emptying and act on hypothalamic receptors to increase satiety and reduce appetite, leading to reduced caloric intake [7, 8].

Chronic low back pain in SEL poses an additional challenge. Opioids are often undesirable in obese patients because of side effects and dependency risk. Medical cannabis has gained attention as an adjunctive analgesic, providing modest pain relief and facilitating opioid sparing and improved function [9].

**Figure 1:**
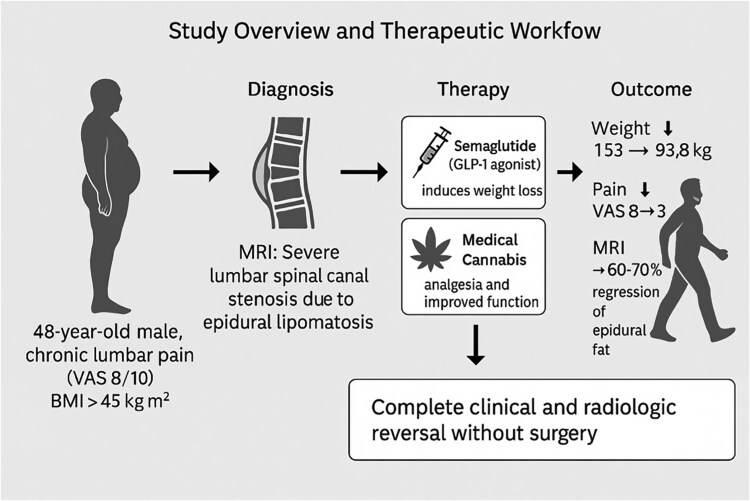
Study overview and therapeutic workflow leading to complete reversal of spinal epidural lipomatosis. This schematic summarizes the diagnostic pathway, therapeutic strategy, and clinical outcomes in a 48-year-old man with severe lumbar spinal canal stenosis caused by spinal epidural lipomatosis.

Here, we describe a case of severe lumbar SEL in a morbidly obese patient successfully managed without surgery. Combined GLP-1 agonist-induced weight loss and medical cannabis-assisted analgesia led to complete clinical and radiological resolution of SEL and obviated the need for decompressive surgery (Fig. 1).

## Case description

A 48-year-old man presented with chronic low back pain that had progressively worsened over several years. His history was notable for morbid obesity (153 kg, body mass index ~46 kg/m2) without chronic steroid use, endocrinopathy or prior spine trauma. He reported exercise intolerance due to back and leg pain, but neurological examination showed full strength, intact sensation, normal reflexes and no bladder or bowel dysfunction.

Lumbar magnetic resonance imaging (MRI) demonstrated diffuse epidural fat from L3 to S1 consistent with SEL, with severe thecal sac compression and maximal canal narrowing at L5–S1 as seen in Fig. 1. Initial conservative management with physiotherapy, lifestyle-based weight loss advice, and non-opioid analgesics produced little benefit. Baseline pain was rated 8/10, and he could ambulate less than 500 m before stopping because of neurogenic claudication.

Given refractory symptoms and marked obesity, aggressive medical weight loss was initiated. The patient started subcutaneous semaglutide, titrated to 2.4 mg once weekly. In parallel, medical cannabis oil was introduced in low doses and titrated as an adjunct for pain control and to avoid opioids with the goal of improving tolerance for physical activity and physiotherapy.

The response was striking. Over seven months, the patient lost approximately 59 kg, reaching 93.8 kg, corresponding to nearly 39% total body-weight reduction (Fig. 3). Semaglutide was well tolerated apart from transient, mild nausea during dose escalation. Cannabis therapy provided satisfactory analgesia without sedation or cognitive blunting, enabling more intensive participation in exercise and rehabilitation. His back pain decreased to around 3/10, and his functional capacity improved from sedentary to walking up to ~24 000 steps per day.

**Figure 2:**
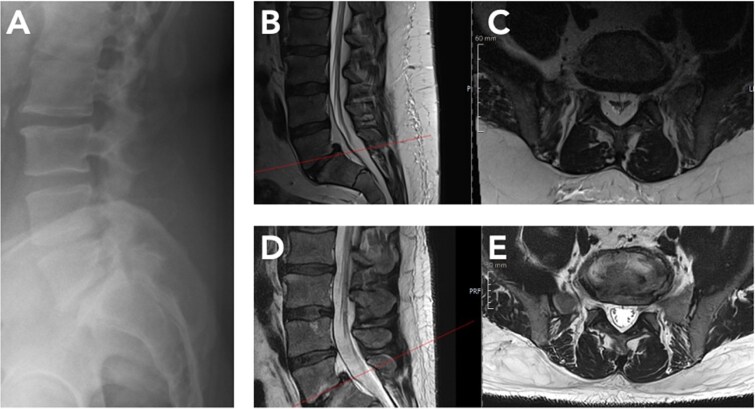
Radiological regression of lumbar spinal epidural lipomatosis (L5/S1) between October 2024 and September 2025. (A) Lateral lumbar spine radiograph (October 2024) showing preserved vertebral alignment without instability but indirect signs of reduced dorsal epidural space at L5/S1. (B, C) Baseline MRI (October 2024). Sagittal and corresponding axial T2-weighted sequence at L5/S1 level demonstrates marked epidural fat accumulation at L5/S1 with compression of the thecal sac. (D, E) Follow-up MRI (September 2025) after massive GLP-1–induced weight loss. Sagittal and corresponding axial T2-weighted image at L5/S1 level shows substantial reduction of epidural fat with re-expansion of the thecal sac.

At eleven months, repeat lumbar MRI showed marked regression of epidural lipomatosis (Fig. 2). Epidural fat volume at L3–S1 had decreased substantially, and the previously severe central canal stenosis had resolved, with normalization of thecal sac contour and canal dimensions. No new degenerative changes were detected. Clinically, the patient reported complete resolution of neurogenic claudication and only mild intermittent low back discomfort. In view of the radiologic and clinical improvement, decompressive surgery was deemed unnecessary.

**Figure 3:**
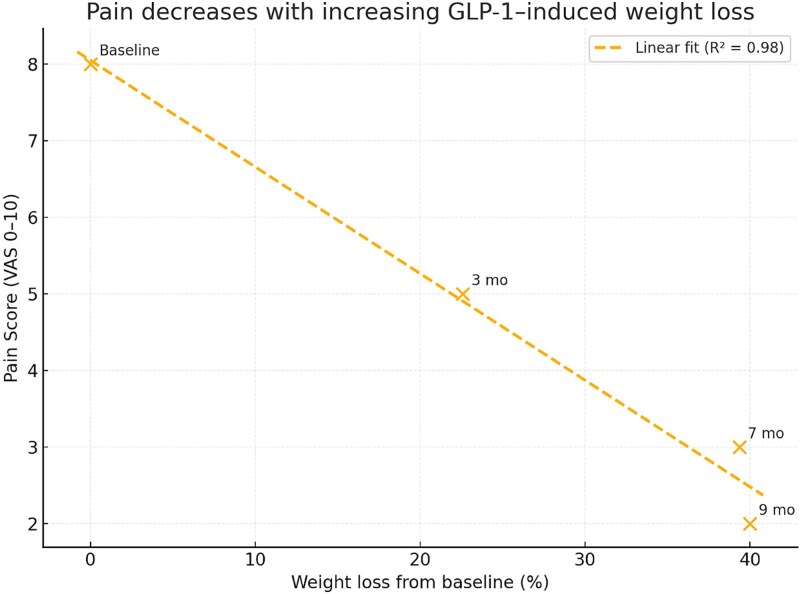
Inverse correlation between body weight and pain intensity during GLP-1–induced weight loss. Scatter plot demonstrating the strong negative correlation between body weight and pain score (VAS) throughout the patient’s clinical course. Each point represents a follow-up time (baseline, 3 months, 7 months, 9 months). Linear regression shows a significant inverse relationship (R^2^ = 0.98), indicating that progressive weight reduction following semaglutide (GLP-1 agonist) therapy was associated with substantial pain improvement.

## Discussion

This case illustrates successful non-surgical management of severe lumbar SEL through pharmacologically assisted weight loss combined with adjunctive cannabis-based analgesia. Although SEL is often regarded as an indication for decompressive surgery once significant stenosis is present, our patient’s course shows that, in the absence of acute neurological deficits, intensive conservative therapy targeting the underlying obesity can lead to both symptomatic and radiologic resolution.

Obesity was the most plausible driver of SEL in this patient. Previous reports have documented reversal of SEL with nonsurgical weight reduction and after bariatric surgery, suggesting that epidural fat is dynamic and responsive to negative energy balance [5, 10–13]. This case extends those observations by demonstrating near-complete reversal of SEL after extreme weight loss achieved with a GLP-1 receptor agonist rather than surgery.

Semaglutide and other GLP-1 agonists are appealing for high-risk spine patients with morbid obesity. They offer a non-invasive means to reduce surgical risk preoperatively and, as shown here, may in some cases eliminate the indication for surgery by treating the causative adiposity. The favorable safety profile observed in large obesity trials aligns with our patients experience [14, 15].

Effective pain management was equally important to break the cycle of inactivity and weight gain. Medical cannabis, introduced as an opioid-sparing strategy, allowed adequate pain control without opioids and facilitated a marked increase in physical activity. While evidence for cannabis in chronic low back pain is still evolving and benefits are generally modest, it can be a useful adjunct within multimodal pain management when conventional analgesics are ineffective or poorly tolerated [16, 17].

Avoiding surgery was advantageous. Morbidly obese patients undergoing lumbar decompression face elevated perioperative risks. Our patient was spared these risks and the recovery period associated with laminectomy. Nonetheless, conservative management of SEL is appropriate only when patients are neurologically intact. Acute cauda equina syndrome, progressive weakness, or intractable pain despite maximal conservative measures still mandate timely surgical decompression [18–20].

## Conclusion

In this case of severe lumbar SEL associated with morbid obesity, combined GLP-1 receptor agonist therapy and medically supervised cannabis use led to major weight loss, complete radiologic regression of epidural lipomatosis, and full resolution of spinal canal stenosis without surgery. This experience supports an intensified, multidisciplinary conservative strategy in neurologically intact obese SEL patients, leveraging modern anti-obesity pharmacotherapy and individualized pain management before resorting to operative intervention.
